# Identification of a Novel 4-gene Prognostic Model Related to Neutrophil Extracellular Traps for Colorectal Cancer

**DOI:** 10.5152/tjg.2024.24131

**Published:** 2024-11-01

**Authors:** Junwen Qian, Jiyun Duan, Dong Cao

**Affiliations:** 1 Department of Gastrointestinal Surgery, Affiliated Hospital of Shaoxing University (The Shaoxing Municipal Hospital), Shaoxing, China; 2 Department of Breast Thyroid Head and Neck Surgery, Affiliated Hospital of Shaoxing University (The Shaoxing Municipal Hospital), Shaoxing, China

**Keywords:** Neutrophil extracellular traps, colorectal cancer, prognostic, immune profiles, drug sensitivity

## Abstract

**Background/Aims:**

Colorectal cancer (CRC) is a significant global health concern, and understanding the molecular mechanisms underlying CRC progression and prognosis is crucial. Neutrophil extracellular traps (NETs) have been implicated in various cancers, but their role in CRC and its clinical implications remain to be elucidated.

**Materials and Methods:**

Transcriptomic data from TCGA of CRC patients were analyzed to assess NETs enrichment and “NETs formation” pathway scores in NETs_high and NETs_low groups. Univariate Cox regression was used to identify prognosis-associated genes with the Log-Rank test for selection. Patients in the TCGA database were randomly split into training and testing sets to build a prognostic model with LASSO Cox regression. Model diagnostic performance was evaluated using Kaplan–Meier curves and receiver operating characteristic analysis. Single-sample gene set enrichment analysis (ssGSEA) was used to determine the abundance of 23 immune cells. ESTIMATE was used to calculate ImmuneScore and ESTIMATEScore, characterizing immune features of CRC samples.

**Results:**

The NETs_high group in CRC showed significantly better survival than the NETs_low group. A robust prognostic model based on PRKRIP1, SERTAD2, ELFN1, and LINC00672 accurately predicted patient outcomes. NETs_high samples exhibited a more enriched immune environment with higher immune cell infiltration levels, as well as ImmuneScore and ESTIMATEScore. PRKRIP1, SERTAD2, ELFN1, and LINC00672 were significantly correlated with key immune cell types. Additionally, 18 drugs displayed differential sensitivity between NETs_high and NETs_low groups, with Daporinad and Selumetinib as potential therapeutic options.

**Conclusion:**

Our findings may catalyze the development of personalized treatment modalities and bestow invaluable insights into the intricate dynamics governing CRC progression.

**Main Points**

- The accurate CRC prognostic model consists of PRKRIP1, SERTAD2, ELFN1, and LINC00672.
- Low NETs are associated with a poor prognosis in CRC patients.
- Daporinad and selumetinib, significantly associated with core genes, are identified as promising therapeutic drugs.
- SERTAD2 is positively correlated with the “neutrophil extracellular trap formation” pathway.

## Introduction

Colorectal cancer (CRC) is a widespread malignant tumor that significantly impacts global health, leading to high incidence and mortality rates.^1^ Colorectal cancer accounts for 11% of all cancer diagnoses,^2^ representing approximately 10% of common cancers worldwide and cancer-related deaths annually.^3^ Colorectal cancer usually originates from genetic mutations or instability, causing uncontrolled division and proliferation of normal colonic mucosal cells.^4^ Risk factors for developing CRC may include dietary choices, environmental exposures, genetic predisposition, and lifestyle factors.^5,6^ Presently, the primary treatment modalities for CRC involve surgical resection, radiation therapy, chemotherapy, targeted therapy, and immunotherapy.^7^ Despite significant progress in diagnosis and therapy methods, the side effects and drug resistance that occur during the treatment process can hinder patients’ recovery to some extent.^8^ Therefore, to better formulate personalized treatment plans, reduce patient discomfort, and enhance treatment effectiveness, identifying new treatment targets and reliable prognostic markers is a top priority.

Recent research has highlighted the role of neutrophil extracellular traps (NETs) in CRC, offering potential as prognostic indicators.^3^ Neutrophil extracellular traps are complex web-like structures composed of DNA, histones, and granule proteins released by activated neutrophils.^9^ Neutrophil extracellular traps are initially believed to play an immune defense role during infection and inflammation processes by helping to protect the body by capturing and clearing microbes and pathogens.^10^ New studies have revealed their multifaceted involvement in various pathological conditions, including cancer, such as tumor development, angiogenesis, metastasis, and cancer-related thrombosis.^11^ In CRC, NETs have gained attention due to their ability to influence the tumor microenvironment, exacerbate inflammation, and modulate immune responses.^12^ Zhang et al^13^ found that the levels of NETs in peripheral blood increase with CRC progression, and neutrophils from CRC patients are more easy to produce NETs. The NET score risk model constructed by Li et al provides a foundation for better prognosis and treatment outcomes for gastric cancer patients.^14^ However, confirmed NETs-related biomarkers in CRC are still absent. In summary, a comprehensive analysis of NETs in relation to CRC may lead to discoveries.

This study aims to explore the significance of NETs in CRC by examining their impact on immune characteristics, drug sensitivity, and patient prognosis. Firstly, transcriptomic, clinical, and file annotation data for CRC were obtained from The Cancer Genome Atlas (TCGA) database. A list of 207 NETs-related genes was compiled through literature research and GeneCards database searches. Differential analysis, univariate and multivariate Cox analysis, as well as the Least Absolute Shrinkage and Selection Operator (LASSO) analysis, were employed to construct a CRC prognostic model based on 4 NET-related differentially expressed genes (DEGs). The model’s predictive performance was validated. Additionally, bioinformatics analyses were conducted to assess the correlation between NETs_high and NETs_low score groups with the “neutrophil extracellular trap formation” pathway, differences in immune infiltration, drug sensitivity, and immune characteristics of the tumor microenvironment. This study suggested a new perspective for better understanding the role of NETs in CRC, adjusting treatment strategies, and significantly improving patient care.

## Materials and Methods

### Colorectal Cancer Data Retrieval

Colorectal cancer transcriptome dataset (455 samples), clinical data, and file annotations were retrieved from the TCGA database (https://portal.gdc.cancer.gov/) using the keyword “TCGA-COAD.” Furthermore, 2 CRC datasets from the GEO database (https://www.ncbi.nlm.nih.gov/), namely GSE17536 (177 samples) and GSE29621 (65 samples), were obtained. The files from the GEO database were processed to obtain CRC transcriptome data and clinical data, which were used for validation in the prognostic model.

### Neutrophil Extracellular Traps Pathway Score Calculation Based on Colorectal Cancer Samples in The Cancer Genome Atlas

Neutrophil extracellular trap-related genes were obtained through literature research and a search in the GeneCards^15^ database (using the keyword “neutrophil extracellular”). The GSVA program package^16^ in R Version 4.3.2 was used to calculate gene set scores based on CRC patient samples in the TCGA transcriptome data. Subsequently, CRC patient samples were divided into high-scoring samples (NETs_high) and low-scoring samples (NETs_low) based on the median score. The risk scores between the 2 groups were estimated using the survival package in R Version 4.3.2.^17^

### Screening Neutrophil Extracellular Trap-related Differentially Expressed Genes in Colorectal Cancer

Based on the grouping into NETs_high vs. NETs_low, NETs-related DEGs in CRC were selected using the limma package in R Version 4.3.2,^18^ with filtering criteria of *P*-value < .05 and |log_2_FC| > 0.1. Subsequently, the DEGs were visualized using the ggplot2 package in R Version 4.3.2.^19^

### Screening Prognosis-related Genes in Colorectal Cancer

Cox regression model, which is capable of simultaneously analyzing the influence of multiple factors on survival duration,^20^ was utilized. The survival package in R Version 4.3.2 was employed to conduct a single-factor Cox regression analysis. Genes associated with CRC prognosis were screened based on NETs-related DEGs, with significance defined as *P* < .05.

### Least absolute shrinkage and selection operator (LASSO) Regression for Prognostic Modeling

Based on the selection of genes associated with CRC prognosis, 50% of the samples were randomly chosen as a training set, while the remaining 50% were designated as a testing set. Simultaneously, the GSE17536 and GSE29621 datasets were employed as external testing sets. The glmnet package in R Version 4.3.2,^21^ utilizing the Cox method, was employed for LASSO regression analysis. Genes were selected for building the prognostic model based on lambda.1se. Risk scores for each sample were computed using the risk function, and samples were categorized into high- and low-risk groups based on the median value. Kaplan–Meier (KM) survival curves were constructed using the KM method, and differences between high- and low-risk groups were tested through the Log-Rank test (*P* < .05). Receiver operating characteristic (ROC) curves were plotted to assess the diagnostic value of the model. Finally, the predictive performance of the model was evaluated using the testing set.

### Gene Set Variation Analysis (GSVA) Enrichment Analysis

In the KEGG database,^22^ a search for pathways related to NETs revealed a pathway named “neutrophil extracellular trap formation.” Using the GSVA package in R version 4.3.2, pathway scores for the NETs_high and NETs_low groups were calculated based on the CRC transcriptome data in the TCGA database. The correlation between prognostic model genes and the “neutrophil extracellular trap formation” pathway was assessed.

### Immune Infiltration Analysis

For CRC samples, expression levels of all genes within the sample were arranged in descending order, and enrichment scores for gene sets were calculated at each position. Subsequently, these scores at each position were averaged or weighted, yielding the ssGSEA^23^ score for the sample concerning that gene set. Differences in immune cells between the NETs_high and NETs_low groups were examined using a *t-*test (*P *< .01), and the correlation between differentially expressed immune cells and prognostic genes was determined.

### Estimate Immunization Score

The ImmuneScore and ESTIMATEScore of CRC samples were computed using the EESTIMATE^24^ index based on TCGA transcriptomic data. These scores provide information regarding tumor purity, the presence of stromal cells, and the level of immune cell infiltration within tumor tissues. Subsequently, *t*-tests were performed to assess the differences in ImmuneScore and ESTIMATEScore between the NETs_high and NETs_low groups (*P* < .05).

### Drug Sensitivity Prediction

The oncoPredict^25^ package in R Version 4.3.2 is used for predicting drug sensitivity based on gene expression levels. The calcPhenotype function within this package was employed to predict drug sensitivity for CRC patient samples based on the GDSC2 database (https://osf.io/c6tfx/). Differences in drug sensitivity between the NETs_high and NETs_low groups were examined using a *t*-test, and differential drugs were selected. Pearson correlation coefficients were computed to evaluate the correlation between drugs and the genes of interest. Subsequently, a correlation *t*-test was conducted to identify drugs significantly associated with prognostic genes.

## Results

### Screening Neutrophil Extracellular Trap-related Differentially Expressed Genes in Colorectal Cancer

**Figure 1.**
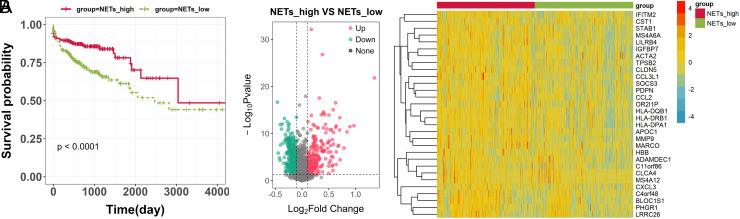
Screening NETs-related DEGs in colorectal cancer (CRC). (A) KM survival analysis of NETs_high and NETs_low groups. (B) Volcano plots (left) and clustered heatmaps (right, top 30 according to |log_2_FC|) of the differentially expressed genes (DEGs) in NETs_high and NETs_low groups.

A total of 207 NETs-related genes were obtained through literature research and a search in the GeneCards database. With 207 NETs-related genes as the background gene set, GSVA enrichment analysis was performed on the transcriptomic data of CRC patients in the TCGA database. According to the median NETs score, the samples were stratified into NETs_high and NETs_low groups. Kaplan–Meier curves revealed that the NETs_high group exhibited a notably superior survival probability compared to the NETs_low group (Figure 1A, *P* < .0001). Besides, the differential analysis revealed 642 DEGs between the NETs_high and NETs_low groups, with 273 genes significantly upregulated and 369 genes significantly downregulated in the NETs_high group (Figure 1B, *P* < .05). The expression patterns of the DEGs further emphasized the distinctive molecular signatures associated with NETs in CRC.

### Screening Prognosis-related Genes in Colorectal Cancer and Constructing Prognostic Modeling

**Figure 2.**
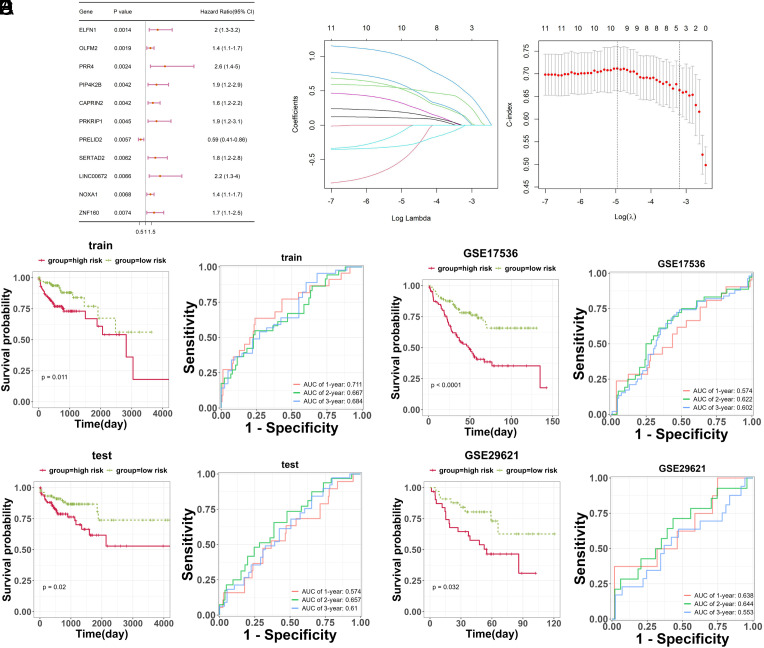
Screening prognosis-related genes in CRC and constructing prognostic modeling. (A) Forest map of prognosis-related genes screened by COX regression algorithm. (B) The relationship curve between LASSO regression coefficients and Lambda (left) and the cross-validation curve (right). (C) The Kaplan–Meier (KM) curve and ROC curve for the training set. (D) The KM curve and ROC curve for the testing set. (E) The KM curve and ROC curve for the GES17536 dataset. (F) The KM curve and ROC curve for the GSE29621 dataset.

Subsequently, 11 CRC prognosis-related genes (NOXA1, PIP4K2B, PRKRIP1, SERTAD2, PRELID2, ZNF160, OLFM2, CAPRIN2, ELFN1, LINC00672, and PRR4) were selected based on the identified 642 DEGs (Figure 2A, *P* < .01). To build a robust prognostic risk model, CRC patient samples from the TCGA database were randomly divided into training and testing sets at a ratio of 1:1. In the training set, the 11 CRC prognosis-related genes were subjected to LASSO Cox regression analysis, and cross-validation was performed using the cv.glmnet function. Finally, 4 selected genes (PRKRIP1, SERTAD2, ELFN1, and LINC00672) were selected and then used to construct the prognostic model (Figure 2B). The prognostic model risk score was calculated as follows: *y* = 0.326894684 × PRKRIP1 + 0.485512958 × SERTAD2 + 0.12480397 × ELFN1 + 0.102761318 × LINC00672.

The diagnostic value of the model was further assessed. The CRC samples were stratified into high- and low-risk groups based on the median risk score of samples in the training set. The high-risk group in the training set exhibited significantly lower survival rates than the low-risk group (*P* = .011, Figure 2C). The ROC curve results indicated good model accuracy at different time points (1-year, 2-year, 3-year) in the training set (AUC values > 0.667, Figure 2C). In the testing set, the high-risk group also exhibited significantly lower survival rates (*P* = .02, AUC value > 0.657, Figure 2D). To further validate the prognostic value of the model in the training set, external validation was performed using the GSE17536 and GSE29621 datasets (Figure 2E and F). The results showed that in both GES17536 (*P* < .0001, AUC value > 0.622) and GSE29621 (*P* = .032, AUC value > 0.644) datasets, the high-risk group suggested significantly lower survival than the low-risk group. This novel model underscored its role in predicting patient outcomes.

### GSVA Enrichment Analysis

**Figure 3.**
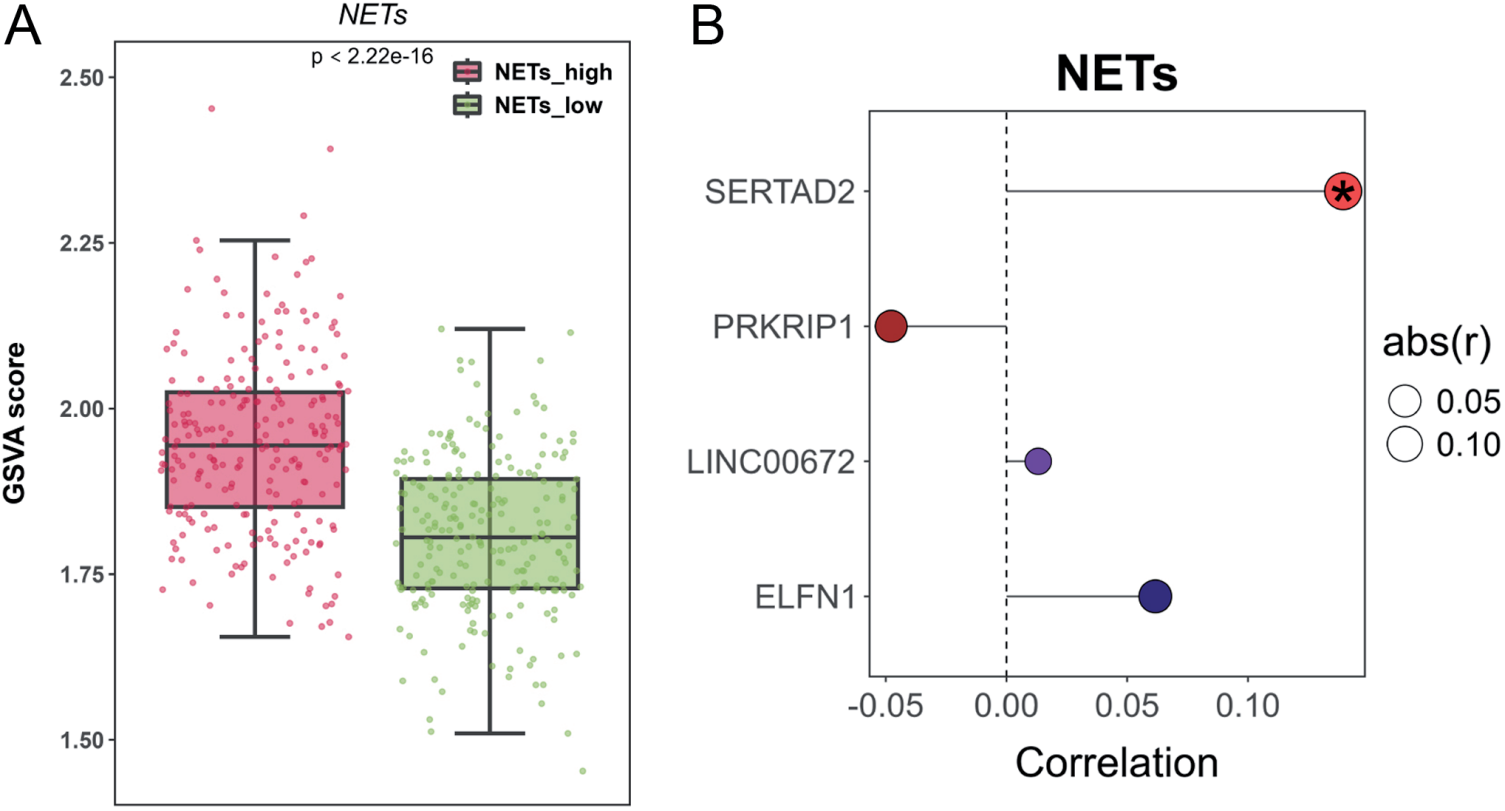
GSVA enrichment. (A) The differences in GSVA scores between NETs_high and NETs_low groups for the “neutrophil extracellular trap formation” pathway. (B) Correlation analysis between 4 prognostic genes (PRKRIP1, SERTAD2, ELFN1, and LINC00672) and the “neutrophil extracellular trap formation” pathway. *P < .05.

To explore the relationship between the identified 4 prognostic genes (PRKRIP1, SERTAD2, ELFN1, and LINC00672) and the “neutrophil extracellular trap formation” pathway, a GSVA enrichment analysis was conducted on the pathway related to NETs based on the TCGA dataset. The analysis indicated that the NETs_high group had significantly higher pathway scores compared to the NETs_low group (Figure 3A, *P* < 2.22 × 10^–16^). Furthermore, a significant positive correlation between SERTAD2 and the “neutrophil extracellular trap formation” pathway was observed (Figure 3B, *P *< .05). This implied that the SERTAD2 gene might play an important role in regulating this pathway.

### Immune Infiltration Analysis and Estimate Immunization Score

**Figure 4.**
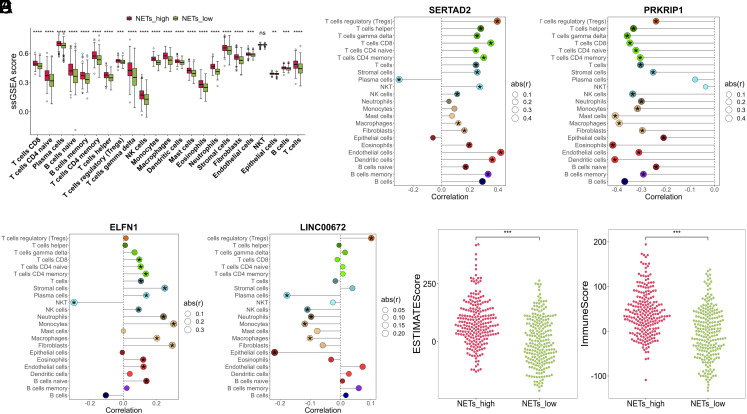
Immune infiltration analysis and ESTIMATE immunization score. (A) The differences in ssGSEA scores between NETs_high and NETs_low groups for the 23 immune cells. (B) Correlation analysis between SERTAD2 and the 23 immune cells. (C) Correlation analysis between PRKRIP1 and the 23 immune cells. (D) Correlation analysis between ELFN1 and the 23 immune cells. (E) Correlation analysis between LINC00672 and the 23 immune cells. (F) The differences in ESTIMATE score (left) and Immune score (right) between NETs_high and NETs_low groups. *P < .05, **P < .01, ***P < .001, ****P < .0001.

Neutrophil extracellular traps are closely linked to the immune microenvironment^26^ and immune cells infiltration might be a predictive indicators in CRC.^27^ Therefore, we examined the differences in the abundance of 23 immune cell types (epithelial cells, mast cells, endothelial cells, stromal cells, fibroblasts, NKT, neutrophils, eosinophils, dendritic cells, monocytes, macrophages, T cells helper, T cells regulatory (Tregs), T cells CD4 naïve, T cells gamma delta, T cells CD4 memory, T cells CD8, NK cells, T cells, Plasma cells, B cells naïve, B cells memory, B cells) between the NETs_high and NETs_low groups (Figure 4A). Apart from NKT cells which had no significant difference between the 2 groups, the ssGSEA scores for the other 22 immune cell types in the NETs_high group were significantly higher than those in the NETs_low group (Figure 4A, *P* < .01), confirming the differences in the immune environment between the NETs_high and NETs_low groups.

Furthermore, we analyzed the correlations between the 23 immune cell types and the 4 prognostic genes (PRKRIP1, SERTAD2, ELFN1, and LINC00672). The results revealed that SERTAD2 and ELFN1 were positively correlated with T cells CD8, T cells CD4 naive, T cells CD4 memory, T cells, stromal cells, NK cells, macrophages, fibroblasts, eosinophils, endothelial cells, and B cells naive, while SERTAD2 was negatively correlated with plasma cells, and ELFN1 was negatively correlated with NKT cells (Figure 4B and D, *P* < .05). Furthermore, PRKRIP1 exhibited negative correlations with most immune cell types except plasma cells and NKT cells (Figure 4C, *P* < .05). LINC00672 was positively correlated with T cells regulatory (Tregs) and negatively correlated with plasma cells, NK cells, neutrophils, monocytes, macrophages, and epithelial cells (Figure 4E, *P *< .05). For a deeper insight into the immune characteristics of CRC samples associated with NETs features, we computed the immune scores for both the NETs_high and NETs_low groups. As shown in Figure 4F, both the ESTIMATEScore and ImmuneScore in the NETs_high group are significantly higher than those in the NETs_low group (*P* < .001). This indicates that the NETs_high group may have a more active immune response and a higher presence of fibroblasts in their tumor microenvironment.

### Drug Sensitivity Prediction

**Figure 5.**
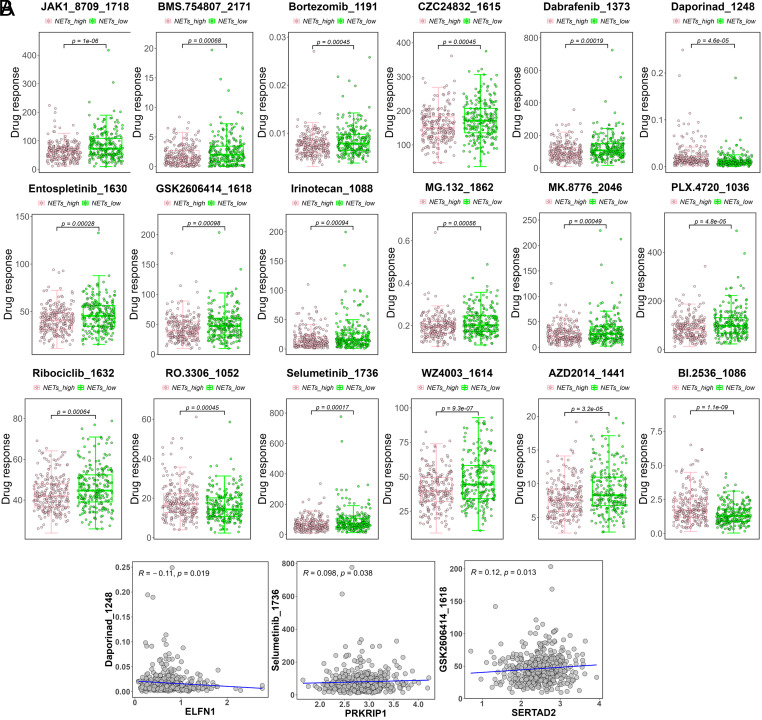
Drug sensitivity prediction. (A) Box plots of sensitivity to 18 drugs (AZD2014_1441, BI.2536_1086, BMS.754807_2171, Bortezomib_1191, CZC24832_1615, Dabrafenib_1373, Daporinad_1248, Entospletinib_1630, GSK2606414_1618, Irinotecan_1088, JAK1_8709_1718, MG.132_1862, MK.8776_2046, PLX.4720_1036, Ribociclib_1632, RO.3306_1052, Selumetinib_1736, and WZ4003_1614) in the high NETs and low NETs groups. (B) Scatterplot of significant correlation between drugs (Daporinad_1248, Selumetinib_1736, GSK2606414_1618) and prognostic genes (ELFN1, PRKRIP1, and SERTAD2), respectively.

In order to gain further insights into drug treatment strategies associated with NETs levels, the variations between the NETs_high and NETs_low groups were evaluated in terms of the sensitivity scores for 18 drugs (AZD2014_1441, BI.2536_1086, BMS.754807_2171, Bortezomib_1191, CZC24832_1615, Dabrafenib_1373, Daporinad_1248, Entospletinib_1630, GSK2606414_1618, Irinotecan_1088, JAK1_8709_1718, MG.132_1862, MK.8776_2046, PLX.4720_1036, Ribociclib_1632, RO.3306_1052, Selumetinib_1736, WZ4003_1614) (Figure 5A). The results demonstrated significant differences in the sensitivity to these 18 drugs between the NETs_high and NETs_low groups. Specifically, the NETs_high group exhibited significantly higher sensitivity to the drugs BI.2536_1086 and RO.3306_1052, while the NETs_low group displayed significantly higher sensitivity to the remaining 16 drugs (*P* < .001).

Subsequently, 3 differentially responsive drugs (Daporinad_1248, Selumetinib_1736, GSK2606414_1618) were selected by calculating the Spearman correlation between the 4 prognosis genes (PRKRIP1, SERTAD2, ELFN1, and LINC00672) and the 18 drugs (Figure 5B). We further verified 2 common drugs, Daporinad_1248 and Selumetinib_1736, through a search on DrugBank (https://www.drugbank.com/). The results revealed significant differences in drug sensitivity based on NETs levels and identified Daporinad_1248 and Selumetinib_1736 as potential responsive drugs through correlation analysis.

## Discussion

Colorectal cancer is one of the most commonly diagnosed and one of the most common cancer-cause death worldwide.^1^ Neutrophils are the most abundant white blood cells and play a crucial role in the immune system, particularly in innate immunity.^28^ In tumors like CRC, NETs have gained significant attention because they may influence tumor growth and development by impacting the tumor microenvironment, exacerbating inflammation, and modulating the immune response.^12^ In this study, it was observed that the NETs_high group exhibited significantly higher survival probabilities compared to the NETs_low group. This suggested that the NETs_high group might have a more active immune response and, as a result, better survival prospects. Subsequent findings further corroborated this hypothesis. The infiltration levels of 22 immune cell types, as well as the ImmuneScore and ESTIMATEScore, were all higher in the NETs_high group. Neutrophil extracellular traps might potentially exert an anti-tumor effect, possibly by triggering immune system activation.^29^ This could impact the tumor’s immune response, subsequently influencing tumor development and patient prognosis.

Understanding the prognosis of CRC is crucial for guiding the direction of selecting appropriate treatment strategies for patients.^30^ Research has identified a correlation between NETs scores and survival rates in various types of cancer, leading to the development of a pan-cancer prognostic marker centered around NETs.^31^ In the present study, a prognostic model for CRC was established based on NETs-related DEGs (PRKRIP1, SERTAD2, ELFN1, and LINC00672). Kaplan–Meier analysis of both a training and a testing set composed of CRC samples demonstrated the excellent predictive performance of this model. Furthermore, external validation through KM curves constructed from the GSE17536 and GSE29621 datasets affirmed the predictive accuracy of the model.

PRKRIP1 encodes a protein typically associated with interferon regulation and antiviral immune responses.^32^ Ozato et al^32^ determined, through bioinformatics methods and immunohistochemistry, that overexpression of PRKRIP1 is an adverse prognostic biomarker for CRC. SERTAD2 is also implicated in tumorigenesis, as it is overexpressed in several cancers.^33^ SERTAD2 is considered to have significant prognostic value in pancreatic ductal adenocarcinoma.^34^ Moreover, the positive correlation between SERTAD2 and the “neutrophil extracellular trap formation” pathway might involve the regulation or participation of the SERTAD2 gene in this biological pathway. However, specific associations require further research for clarification. ELFN1 encodes a protein typically found in the extracellular matrix and is involved in cell adhesion and signaling.^35^ He et al^36^ demonstrated that ELFN1-AS1 promotes CRC’s immune escape from NK cells by facilitating the binding of GCN5 and SND1 to GDF15, considering ELFN1-AS1 as a potential therapeutic target for CRC. This study also verified the positive correlation between ELFN1 and NK cells. LINC00672 is a long-stranded non-coding RNA (lncRNA) associated with many diseases and tumors.^37^ Mendelaar et al^38^ found that mutations in LINC00672 present in CRC patients may affect the efficacy of general treatment methods. Furthermore, PRKRIP1, SERTAD2, ELFN1, and LINC00672 were either positively or negatively correlated with major cell types in 23 immune cell types. This correlation could potentially impact the immune response to tumors and patient prognosis. However, specific mechanisms and biological significance require further in-depth research for detailed explanations.

The differences in the immune environment between the NETs_high and NETs_low groups, as well as the 4 genes used to construct the prognostic model, suggested that immunotherapy might offer more assistance to high-risk patients. For both groups of patients, we selected drugs that were sensitive to their treatment. Subsequently, we further screened drugs significantly associated with prognostic genes (Daporinad_1248 and Selumetinib_1736). Daporinad (also known as RST-001) primarily functions by inhibiting NAD(P)H quinone dehydrogenase 1. It has been observed by Sharior et al in a mouse model of ovarian cancer that treatment with olaparib (a PARP inhibitor) and Daporinad (an NAMPT inhibitor) can deplete intracellular NAD^+^.^39^ This depletion of NAD^+^ leads to the induction of double-strand DNA breaks and promotes apoptosis by caspase-3 cleavage, thereby reducing the development of therapeutic resistance in ovarian cancer.^39^ Selumetinib (Koselugo) is an orally administered selective inhibitor of MEK 1 and 2,^40^ which are components of the MAPK signaling pathway.^41^ Song et al^42^ found that the combination of Selumetinib with hesperetin enhances the inhibitory effects on the MAPK signaling pathway in CRC. These findings can potentially provide more personalized treatment options and improve therapeutic outcomes for CRC patients.

## Conclusion

In conclusion, this study investigated the significance of NETs in CRC and their impact on prognosis. We identified a distinct gene signature using comprehensive analyses and constructed a robust prognostic model based on 4 genes (PRKRIP1, SERTAD2, ELFN1, and LINC00672). The model demonstrated strong predictive power for patient outcomes, which was validated in external datasets. Furthermore, our analysis revealed a link between NETs and immune responses in CRC. NETs_high samples exhibited an enriched immune environment, potentially influencing CRC prognosis. Additionally, we identified 18 drugs with varying sensitivity between NETs_high and NETs_low groups, with 2 promising drugs, Daporinad and Selumetinib, showing potential for personalized treatment. These findings shed light on the complex interplay of NETs, immune responses, and drug sensitivity in CRC, offering insights into better prognosis prediction and tailored therapeutic strategies for CRC patients.

## Data Availability

The data that support the findings of this study are available on request from the corresponding author.
